# Why Dosing Matters: A Closer Look at the Dose–Response Relationship With OnabotulinumtoxinA

**DOI:** 10.1111/jocd.70170

**Published:** 2025-04-26

**Authors:** Wolfgang G. Philipp‐Dormston, John H. Joseph, Jean D. A. Carruthers, John P. Fezza, Mansi Mukherjee, Ahmed Yasin, Maria Musumeci

**Affiliations:** ^1^ Hautzentrum Köln Cologne Germany; ^2^ Faculty of Health University Witten/Herdecke Witten Germany; ^3^ Clinical Testing of Beverly Hills Beverly Hills California USA; ^4^ Department of Ophthalmology University of British Columbia Vancouver British Columbia Canada; ^5^ Center for Sight Sarasota Florida USA; ^6^ Kaya Skin Clinic Dubai UAE; ^7^ Global Aesthetics Medical Affairs Allergan Aesthetics, an AbbVie Company Dubai UAE; ^8^ Global Aesthetics Medical Affairs Allergan Aesthetics, an AbbVie Company Rome Italy

**Keywords:** botulinum neurotoxin, ceiling dose, dose–response, duration, onabotulinumtoxinA

## Abstract

**Background:**

OnabotulinumtoxinA is licensed in many countries for simultaneous treatment of three areas of the upper face: glabellar lines, 20 U; lateral canthal lines, 24 U; and forehead lines, 20 U.

**Aims:**

To assess the onabotulinumtoxinA dosing science and dose–response relationship in the treatment of upper facial lines (UFL).

**Methods:**

Key practical questions are addressed using available data.

**Results:**

OnabotulinumtoxinA doses were selected for Phase 3 registrational trials based on rigorous dose‐ranging studies. In clinical practice, it is important to consider the relationship between dose and efficacy outcomes, duration, and safety. Interstudy comparison of duration analyses is complicated by the lack of a single comprehensive definition, but trial data with standard onabotulinumtoxinA dosing in the glabella suggest a median effect duration of ~4 months. Treatment of UFL at below the approved dose is associated with a shorter duration, inferior response rates, and lower patient satisfaction; there is no evidence that underdosing reduces adverse event risk. It may therefore be advisable to avoid going below the licensed dose unless there is a clear clinical rationale. By contrast, there is growing evidence that treatment outcomes can be further improved using doses above those currently licensed, without adversely affecting safety—as demonstrated in the glabella. Further studies are needed to assess this in lateral canthal and forehead lines. Additional work is also required to examine potential ceiling doses and better understand the dose–response relationship in patient subgroups.

**Conclusions:**

Appropriate dosing of onabotulinumtoxinA is essential for maximizing benefit and ensuring patient satisfaction.

## 1 Introduction

Injectable botulinum neurotoxin formulations (BoNTs) are commonly used in aesthetic medicine [1, 2], and onabotulinumtoxinA (Botox Cosmetic/Vistabel, Allergan Aesthetics, an AbbVie Company, Madison, NJ) is the most widely studied in this setting.

When onabotulinumtoxinA was first used for aesthetic purposes more than 20 years ago, there were only limited data from high‐quality trials to inform decision making around dosing and treatment techniques. However, large numbers of clinical studies were performed in the intervening years. These have given injectors far greater scope for evidence‐based medicine (EBM) in their daily practice—based on rigorously conducted scientific research. Practical experience undoubtedly remains highly valuable, but it can be subject to bias and variability, and hence should be complemented with EBM.

The current onabotulinumtoxinA license for the treatment of glabellar (GL), lateral canthal (LCL), and forehead lines (FHL) [3, 4] is based on data from multiple Phase 3 randomized controlled trials [5, 6, 7, 8, 9, 10, 11].

For simultaneous treatment of all three areas of the upper face, the approved dose in the US and Europe is 64 U (20 U for GL; 24 U for LCL; 20 U for FHL) [3, 4]. In a randomized, placebo‐controlled registration study of 787 subjects, injection of 64 U across these three sites was found to be safe and effective in reducing the severity of lines [11, 12]. Satisfaction levels were high based on validated patient‐reported outcome (PRO) instruments, with a signal for potentially greater improvements when treating all three areas compared with only two [12]. Furthermore, among responders in the Phase 3 program, more than 90% felt that they had received natural‐looking outcomes [13].

In normal daily practice, the dose injected is a matter for clinicians to decide in partnership with their patients, using the available evidence as a component of this process. However, careful consideration should be given to the relationship between dose and outcomes.

In a preclinical analysis of rats injected with escalating doses of onabotulinumtoxinA in the tibialis anterior hindlimb muscles [14], paralyzing effects were assessed using the digit abduction score assay [15]. Below a saturating upper dose, there was a clear positive correlation between dose, peak effect, and duration of action (log‐linear for dose–duration and linear for effect–duration). In other words, the higher the dose, the greater the effect and the longer the duration.

This raises the question of whether such a correlation translates into human patients. Based on recent clinical data [16], some experts now support the more routine use of higher doses. For example, a 2022 Italian consensus statement on onabotulinumtoxinA, updating advice from a paper published 4 years earlier, noted the following: [17] “Compared with our previous consensus, some of the recommended doses have been substantially increased. The goal is to achieve a longer lasting result without increasing the risk of side effects.” Current data show that higher doses do not impact safety outcomes, suggesting that concerns about safety are not warranted.

The purpose of this paper is to consider key questions around the dosing science and current knowledge of the dose–response relationship for onabotulinumtoxinA in the treatment of upper facial lines (UFL). The primary focus is on the impact of dose on effect duration. However, other key outcomes will also be considered—including response rates, PROs (an essential indicator of success in aesthetic medicine), and adverse events (AEs).

## 2 How Does BoNT Duration Relate to the Mechanism of Action?

BoNTs paralyze target muscles through the inhibition of neurotransmitter release at peripheral cholinergic nerve terminals [18, 19]. The pharmacologically active 150‐kDa BoNT molecule consists of a 50‐kDa light chain connected via a disulfide bond to a 100‐kDa heavy chain [19]. There are at least seven different serotypes, known as BoNTs A–G. However, serotype A (BoNTA) formulations are the most widely used for therapeutic and aesthetic purposes, owing to their extended duration of action.

The biological processes underlying the activity of BoNTs are: (1) heavy chain‐mediated binding to nerve terminals, primarily via protein–protein and protein–glycan interactions with glycosylated synaptic vesicle glycoprotein 2 (SV2) receptors; (2) internalization within an endocytic compartment; (3) membrane translocation and release of the light chain into the cytosol by reduction of the disulfide bond linking it to the heavy chain; and (4) light‐chain metalloprotease cleavage of soluble N‐ethylmaleimide‐sensitive factor attachment protein receptor (SNARE) proteins, leading to inhibition of neurotransmitter release [18, 19, 20].

Studies are increasingly elucidating the molecular mechanisms that underlie the duration science of BoNTs. For example, glycosylation of SV2 is required for BoNTA binding and uptake [20] but glycan profiles can vary according to sex and age [21], suggesting that patient‐related factors could have a significant effect. There may also be important age‐related morphological changes at motor endplates [22], and the impact of this process on BoNTA effect and duration warrants further study.

Within the neuron, a stabilizing interaction between the light chain of serotype A BoNTs and cytoskeletal septin proteins appears to protect the light chain from degradation and may be fundamental to the persistence of the toxin [23]. Delivery of recombinant BoNTA light chain to differentiated PC12 cells led to dose‐dependent cleavage of the SNARE component SNAP‐25, and BoNTA exposure was also associated with dose‐dependent clustering of septin at the plasma membrane. The shorter duration of effect of some other BoNT serotypes relative to serotype A may be at least partly due to a lack of interaction with septins [23].

Recent studies in rats have suggested that the neurogenic regulator, agrin, may play an important role in toxin‐triggered nerve sprouting, which helps to restore blocked muscle contraction and thus limits the duration of action of BoNTs [24]. Targeting this function with anti‐agrin antibodies could play a role in future efforts to delay the recovery of muscle strength and thus prolong the effects of BoNTAs.

Further work will also be required to better understand how these various mechanisms affect the duration of BoNTA formulations—and indeed their broader efficacy and safety.

## 3 How Were the Approved OnabotulinumtoxinA Doses Derived?

As biological products, the potency of BoNTAs cannot be measured in mass units (e.g., nanograms) because these assess protein quantity but not necessarily activity [25, 26]. Hence, there is no mention of mass units in BoNTA package inserts, with vial contents and potency instead expressed using biological units. In this context, 1 U is the amount of BoNTA that kills 50% of mice when injected intraperitoneally [26, 27].

These units are not interchangeable between BoNTAs, owing to differences in the complex manufacturing processes of each product (e.g., master cell banks, excipients, and purification methods), differences in accessory proteins, and variations in the assays used to assess unit dosing (e.g., animal strain/sex/age, diluents, and the reference standards employed) [25, 26]. Thus, regulators across the world have stated that the biological activity units of one BoNTA product cannot be compared with or converted into those of any other [25].

In the early days of BoNTA use for aesthetic purposes, the doses and dilutions employed were selected based largely on expert opinion. It was then essential to assess these parameters more scientifically. The unit doses of onabotulinumtoxinA evaluated in registrational Phase 3 trials (and subsequently licensed) were not arrived at arbitrarily but had their basis in rigorously conducted dose‐ranging studies (Table 1).

**TABLE 1:** Key onabotulinumtoxinA dose‐ranging studies in UFL indications.

Study design	Population	Doses assessed	Key effectiveness outcomes	Safety
*Glabellar lines*				
Double‐blind, randomized dose‐ranging trial [28]	80 females with moderate‐to‐severe GL	10, 20, 30, 40 U	Longer duration of effect, higher response rate, and greater maximum improvement with 20–40 U versus 10 U Four‐month relapse rate:^a^ 10 U, 83%; 20 U, 33%; 30 U, 30%; 40 U, 28% (*p* ≤ 0.0017)	No significant association between dose and AEs
Double‐blind, randomized trial [29]	142 Japanese patients with moderate‐to‐severe GL	10, 20 U, placebo	Week 8 investigator‐assessed response rate:^b^ 10 U, 81.8%; 20 U, 68.2%; placebo, 0% (*p* < 0.001 for 10 or 20 U vs. placebo) Week 8 patient‐assessed response rate:^c^ 10 U, 70.5%; 20 U, 88.6%; placebo, 0% (*p* = 0.035 for 20 U vs. 10 U)	No significant association between dose and AEs
Double‐blind, randomized dose‐ranging trial [30]	80 males with moderate‐to‐severe GL	20, 40, 60, 80 U	Investigator‐assessed response rate:^b^ 20 U, 65%; 40 U, 90%; 60 U, 95%; 80 U, 100% (*p* < 0.0001) Mean time to relapse:^a^ 20 U, 17.6 weeks; 40 U, 21.7 weeks; 60 U, 22.8 weeks; 80 U, 24.2 weeks	No significant association between dose and AEs
Double‐blind, randomized trial [16]	226 females with moderate‐to‐severe GL	20, 40, 60, 80 U, placebo	Week 24 investigator‐assessed response rate:^d^ 20 U, 16.0%; 40 U, 32.0%; 60 U, 30.6%; 80 U, 38.5%; placebo, 0% (*p* < 0.05 for 40 and 80 U vs. 20 U) Mean duration of response:^e^ 20 U, 19.7 weeks; 40 U, 24.1 weeks; 60 U, 24.1 weeks; 80 U, 24.0 weeks (*p* < 0.05 for higher doses vs. 20 U) Week 24 patient satisfaction was higher with 40, 60, and 80 U versus 20 U (*p* < 0.05)^f^	No significant association between dose and AEs
*Lateral canthal lines*				
Double‐blind, randomized, dose–response study [31]	162 patients with moderate‐to‐severe LCL	3, 6, 12, or 18 U per side, or placebo	Day 30 response rate:^d^ 3 U, 45.5%; 6 U, 51.5%; 12 U, 87.1%; 18 U, 84.8%; placebo, 15.6% (*p* < 0.05 for all groups vs. placebo) Duration of effect:^e^ 3 U, 36 days; 6 U, 92 days; 12 U, 120 days; 18 U, 112 days Greater effect on patient satisfaction with higher doses (FLTS)	No significant association between dose and AEs
*Forehead lines*				
Double‐blind, randomized dose‐ranging trial [32]	175 patients with moderate‐to‐severe FHL	10 or 20 U (plus 20 U for GL), placebo	Day 30 investigator‐assessed response rate:^b^ 10 U, 86.4%; 20 U, 91.2%; placebo, 1.7% (*p* < 0.001 for 10 and 20 U vs. placebo) Duration of effect:^g^ 10 U, 113.0 days; 20 U, 118.5 Day 30 patient satisfaction (response rate on FLO‐11) was greater with 10 or 20 U versus placebo (*p* < 0.001)	No significant association between dose and AEs
*Upper facial lines (GL, LCL, and FHL together)*				
Double‐blind, randomized, dose‐comparison study [33]	60 female patients with moderate‐to‐severe UFL	32, 64, 96 U	Response rate:^h^ 32 U, 26%; 64 U, 61%; 96 U, 71% (*p* = 0.0007) Significantly greater duration of effect^h^ with higher doses (*p* < 0.0001)	No significant association between dose and AEs

Abbreviations: AE, adverse event; FHL, forehead lines; FLO‐11, Facial Line Outcomes‐11; FLTS, Facial Lines Treatment Satisfaction questionnaire; GL, glabellar lines; LCL, lateral canthal lines; UFL, upper facial lines.

^a^ Return to baseline value on the Facial Wrinkle Scale at two consecutive visits.

^b^ Improvement in severity to “none” or “mild.”

^c^ On a 9‐point scale.

^d^ ≥ 1‐grade improvement from baseline.

^e^ Time to return to baseline severity in responders.

^f^ Proportion of responders stating that they were “mostly” or “very” satisfied with various aspects of the treatment effect using the Facial Line Satisfaction Questionnaire.

^g^ Loss of severity rating of “none” or “mild.”

^h^ Score of ≥ 1 on the 9‐point patient global assessment scale.

For example, in the treatment of GL, a double‐blind, randomized trial assessed onabotulinumtoxinA at doses of 10, 20, 30, or 40 U in 80 female patients with moderate‐to‐severe GL [28]. The 20, 30, and 40 U doses were all found to be significantly more effective than 10 U, with higher response rates and longer durations of effect. The 4‐month relapse rate, assessed based on reversion to baseline score on the Facial Wrinkle Scale (FWS), was significantly greater with 10 U compared to larger doses (83% vs. 28%–33%, respectively; *p* ≤ 0.0017). Moreover, patient satisfaction was typically greater with higher doses, although the differences were not statistically significant.

An Asian study also provided support for the 20 U glabellar dose. This was a double‐blind, randomized trial comparing onabotulinumtoxinA 10 U or 20 U versus placebo for treating moderate‐to‐severe GL in 142 Japanese subjects [29]. Physician‐assessed response rates were numerically higher throughout follow‐up with 20 U versus 10 U. In addition, the patient‐assessed response rate was significantly greater at Week 8 with the higher dose (20 U, 88.6%; 10 U, 70.5%; *p* = 0.035).

**FIGURE 1:**
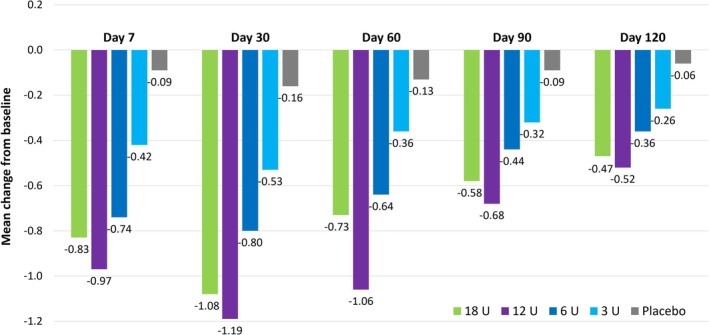
Mean change from baseline in investigator‐assessed LCL severity at maximum smile in a dose‐ranging study of onabotulinumtoxinA. Study subjects were randomized to bilateral treatment on Day 0 with one of four onabotulinumtoxinA doses or placebo [31]. LCL severity was assessed for each side separately on a four‐point scale. *p* < 0.05 for onabotulinumtoxinA versus placebo from Day 7 to Day 120 for the 6 U, 12 U, and 18 U doses, and from Day 7 until Day 30 for the 3 U dose. LCL, lateral canthal lines.

For LCL treatment, the key analyses came from a double‐blind, randomized, dose–response study of 162 patients with moderate‐to‐severe LCL injected with onabotulinumtoxinA at doses of 3, 6, 12, or 18 U per side [31]. There were dose‐dependent improvements in the magnitude and duration of effect, with no clear difference between the two highest doses (Figure 1). For example, the estimated median times to return to baseline LCL severity were 36, 92, 120, and 112 days for the 3, 6, 12, and 18 U doses, respectively. These data provided the rationale for using the 12 U dose in registrational studies. Although there appeared to be a small decrease in duration with 18 U versus 12 U, this was not statistically significant; in our clinical experience, the higher dose is associated with an effect duration at least as long as the approved dose of 12 U.

**FIGURE 2:**
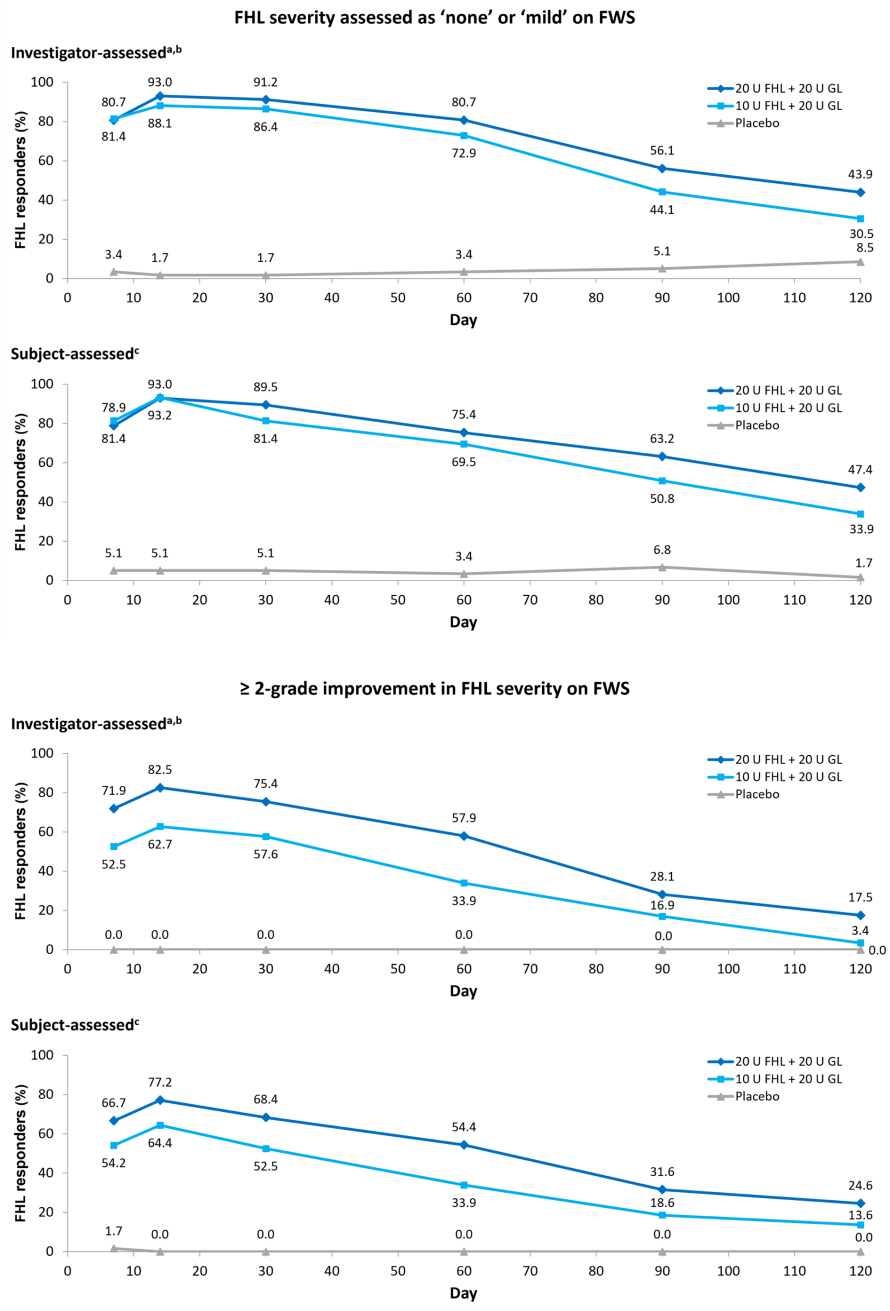
Improvement in FHL following onabotulinumtoxinA treatment as assessed on FWS by investigators and study subjects. Patients were randomized to treatment with 10 or 20 U of onabotulinumtoxinA in the forehead, each combined with 20 U in the glabella (thus 30 or 40 U in total), or with placebo [32]. Assessments were made at maximum eyebrow elevation. ^a^ *p* ≤ 0.05 through Day 120 (active arms vs. placebo). ^b^ *N* values were 59, 57 and 59 in the 30 U, 40 U, and placebo groups, respectively. ^c^ *p* ≤ 0.001 through Day 120 (active arms vs. placebo). FHL, forehead lines; FWS, Facial Wrinkle Scale; GL, glabellar lines.

For treatment of FHL, a double‐blind, randomized, dose‐ranging study was performed in 175 patients with moderate‐to‐severe FHL [32]. Participants were injected with 10 U or 20 U of onabotulinumtoxinA in the forehead (each combined with 20 U in the glabella), or with placebo. The 20 U forehead dose was associated with a trend toward greater response rates and longer duration of effect compared with 10 U. For example, investigator‐assessed response rates at Day 30 were 91.2% and 86.4%, respectively, based on patients achieving “none” or “mild” on FWS at maximum eyebrow elevation (Figure 2A). These proportions continued to show separation through later timepoints out to Day 120 [32]. Moreover, the proportions of patients with ≥ 2‐grade improvement on FWS was also numerically greater with the 20 U forehead dose compared with 10 U across the first 120 days post‐treatment (Figure 2B) (data on file, Abbvie Inc.).

Finally, for simultaneous treatment of multiple areas, a double‐blind, randomized, dose‐comparison study was performed in 60 female patients treated for all three types of UFL with 32, 64, or 96 U of onabotulinumtoxinA [33]. The response rate was significantly increased at higher doses, based on patient global assessment of improvement: 26% with 32 U; 61% with 64 U; and 71% with 96 U (*p* = 0.0007). The duration of effect was also longer with higher doses (*p* < 0.0001).

Importantly, none of these studies found any impact of dose on safety outcomes—and there was no apparent decrease in AE frequency, severity, or duration when using doses below licensed levels.

Overall, this work underlaid the dose selection process for registrational studies. It also afforded some preliminary signals of potential to improve efficacy using higher doses, without impacting safety outcomes.

## 4 Has the Duration of Effect of OnabotulinumtoxinA Been Analyzed in Clinical Studies?

There is no universal definition for measuring the clinical duration of effect of BoNTAs, and it can vary significantly depending on the evaluation method used. Some studies have analyzed duration according to the time at which patients lose “responder” status—for example, loss of ≥ 2‐ or ≥ 1‐point improvement on a response assessment instrument such as FWS—whereas others have instead used the time at which lines return to baseline severity. The latter definition will of course yield a longer measurement of duration than the former.

In addition, responder rates also vary according to the definition used and this can impact on how duration is perceived. Indeed, as shown in Figure 2, assessment of response in the same dataset using different criteria (“none or mild” vs. “≥ 2‐point improvement” on FWS) gave quite distinct results in an onabotulinumtoxinA dose‐ranging study.

Clinicians must therefore be cautious when comparing results from different studies and attempting to apply them in routine practice.

The duration of clinical effect with onabotulinumtoxinA was estimated in a meta‐analysis of four randomized controlled trials of patients treated for GL using a 20 U dose [34]. Here, the calculation was based on maintaining a response (“none” or “mild” on FWS) at maximum contraction. Kaplan–Meier analysis of pooled data suggested a median duration of effect of 120 days (95% confidence interval: 119–121).

Injectors must always bear in mind that the duration of effect in individual patients may vary according to a variety of factors, including sex, age, the area injected, the severity of the lines, and previous treatment [35]. From a practical perspective, duration will naturally vary around the median, with “outliers” on both sides of the curve. Thus, small proportions of patients may have particularly short response times while some others will have longer durations of response lasting 6 months or more.

In our experience, the effect duration of onabotulinumtoxinA is typically longer when all three types of UFL are treated simultaneously, as compared with injecting one or two areas in isolation. This has not yet been fully demonstrated in clinical studies, although a Phase 3 trial showed incremental improvements in both response rate and PROs when treating three versus two areas in the upper face [11, 12]. These are mimetic muscles, and balancing their effects is crucial to brow position and shape; concurrent treatment of the depressor and elevator musculature may help to maintain the balance and thus optimize outcomes [36]. However, further study is needed to confirm this point.

## 5 Is There any Evidence That Higher Doses Can Improve Efficacy and Duration of Response?

Various studies of GL treatment have suggested that high doses of BoNTA (above those currently licensed) can improve the duration of effect without significantly increasing the risk of AEs [37]. Specifically, with onabotulinumtoxinA, at least two publications have shown that increasing the GL dose beyond 20 U could yield longer‐lasting responses—along with improvements in other measures of effectiveness, such as response rates and patient satisfaction [16, 30].

The first was an early randomized study assessing the use of 20, 40, 60, or 80 U of onabotulinumtoxinA in 80 male patients with moderate‐to‐severe GL [30]. A clear dose effect was observed (Table 1). For example, the proportion of patients with an improvement in GL severity from “severe” to “mild” or “none” increased with dose, from 65% in the 20 U group to 90% with 40 U, 95% with 60 U, and 100% with the 80 U dose (*p* < 0.0001). The mean time to relapse also appeared to increase—from 17.6 weeks with 20 U up to 24.2 weeks with 80 U.

The second analysis was from a 48‐week, randomized, double‐blind study comparing higher doses of onabotulinumtoxinA (40, 60, or 80 U) versus the on‐label 20 U dose or placebo for treating moderate‐to‐severe GL in 226 female patients [16]. Week 24 responder rates (the primary endpoint) were 0% with placebo, 16% with 20 U, and over 30% with 40, 60, or 80 U (*p* < 0.05 for 40 and 80 U vs. 20 U). In this study, duration of response was assessed based on the time to return to baseline GL severity at maximum frown among Week 4 responders. The median duration was longer with all three of the higher doses compared with 20 U: 24.0–24.1 weeks versus 19.7 weeks, respectively (*p* < 0.05). Patient‐reported treatment satisfaction was also improved with higher onabotulinumtoxinA doses. Indeed, at Week 24, the proportion of responders stating that they were “mostly” or “very” satisfied with various aspects of the treatment effect was higher in each of the 40, 60, and 80 U groups compared with 20 U (*p* < 0.05).

Importantly, this study administered onabotulinumtoxinA at a higher concentration than that specified in the product label [3, 4]. The aim was to reduce the injection volume and mitigate potential AEs associated with localized BoNTA diffusion, which may be decreased with lower volumes [38]. The impact of using a more concentrated onabotulinumtoxinA solution on effect duration and other efficacy measures remains to be fully elucidated, and further studies are required. However, it is possible that outcomes could be improved by exposing the targeted muscles to a larger local dose without increasing the number of units given overall [39].

## 6 Is There a Ceiling Dose?

The concept of a BoNTA “ceiling” dose—above which further increases yield no additional benefit—was first analyzed as long ago as 1996 [40]. In that study, Sloop and colleagues injected onabotulinumtoxinA at seven different doses (1.25, 2.5, 5.0, 7.5, 10, 15, or 20 U) into the extensor digitorum brevis (EDB) muscles of 13 healthy adults and assessed the dose–response relationship using electromyography. The data showed a maximal paralysis of around 85%–90% at 7.5 U, with the curve then plateauing and little additional effect achieved using higher doses [40].

In the treatment of GL, a 2005 dose‐ranging study of onabotulinumtoxinA 20–80 U in male patients found that response rates and duration of effect continued to improve as the dose increased, and there was no clear ceiling dose [30]. By contrast, a 2022 study in females suggested a ceiling at around 40 U, with limited or no further improvements in efficacy outcomes above this dose [16]. Thus, any potential ceiling dose may differ between males and females and could also vary by indication. Other factors that may influence the ceiling include patient age and muscle mass [37].

Further studies are required to understand the mechanistic basis for the flattening of the dose–response relationship and to better define ceiling doses. This work should focus not only on investigator assessments but also on PROs.

## 7 Does Increasing the Dose of OnabotulinumtoxinA Affect Safety Outcomes?

A recent review of five GL studies based on high doses of various different BoNTAs (including onabotulinumtoxinA) observed no apparent increase in AE rates compared with licensed doses [37]. Moreover, studies specifically assessing onabotulinumtoxinA at between 10 U and 80 U in the treatment of moderate‐to‐severe GL found no dose–response relationship in the development of treatment‐emergent AEs [16, 28, 29, 30]. For example, in a recent trial, there was no apparent dose effect on the incidence or severity of any category of AEs at doses of 20–80 U [16]. Among 208 onabotulinumtoxinA‐treated patients in this study, only 1 experienced eyelid ptosis (mild in severity; 80 U group) and 1 showed eyebrow ptosis (20 U group).

Similarly, no dose effect on AEs was observed in dose‐ranging studies of onabotulinumtoxinA in the treatment of LCL or FHL [31, 32], or when injecting all three approved UFL areas in the same session [33].

Cumulative dosing with repeated treatment also appears not to affect safety outcomes. In a recent meta‐analysis of double‐blind placebo‐controlled onabotulinumtoxinA trials, a maximum cycle analysis found no evidence of elevated risk with increasing cumulative dose [41]. Furthermore, in a real‐world analysis of 194 patients injected regularly with onabotulinumtoxinA for GL (and other UFL) over a mean period of 9.1 years, AEs were typically mild, infrequent, and declined in number over time [42, 43].

There is an important question around the potential to increase the risk of neutralizing antibody (NAb) development when using higher doses of BoNTA. However, a 2023 meta‐analysis of 33 longitudinal clinical studies in aesthetic and therapeutic indications found that NAb incidence rates were low with onabotulinumtoxinA [44]. Twenty‐seven of 5876 evaluable patients (0.5%) developed NAb after treatment, and only 16 (0.3%) remained NAb‐positive at study exit. Furthermore, among 1725 individuals treated for aesthetic indications (GL or LCL) for up to five treatment cycles, none had NAbs at study exit. This analysis also found no clear correlation between NAb development and patient or treatment characteristics (e.g., gender, indication, dose level, dosing interval, treatment cycles, or injection site) [44].

From a practical perspective, even the highest BoNTA doses used in aesthetic medicine are substantially lower than those injected in adult therapeutic indications. Thus, the risk of NAb development is unlikely to be of great clinical relevance during the treatment of UFL. Moreover, by using the appropriate dose to achieve the best possible efficacy and duration, injectors can reduce the need for more frequent repeated dosing—which might contribute to lowering the overall risk of NAb formation.

## 8 Conclusions

Key takeaway messages relating to onabotulinumtoxinA duration and dosing science are summarized in Table 2. The molecular mechanisms that underlie this are increasingly well understood.

**TABLE 2:** OnabotulinumtoxinA duration and dosing science: summary.

Question	Key takeaway
How does BoNT duration relate to the mechanism of action?	Studies are now revealing the molecular mechanisms that underlie BoNT duration (e.g., SV2, glycosylation, septin interaction, agrin function, etc.) but further work is still needed [20, 23, 24]
How were the approved onabotulinumtoxinA doses derived?	The doses used in registrational trials (and subsequently licensed) were not arbitrary but rather had their basis in rigorously conducted dose‐ranging studies [28, 31, 32, 33]
Has the duration of effect of onabotulinumtoxinA been analyzed in clinical studies?	There is no universal definition for assessing clinical duration of effect—and it can vary significantly depending on the definition used Nonetheless, trial data with the standard onabotulinumtoxinA dose of 20 U for GL suggest a median duration of effect of ~4 months [34] In our experience, the effect duration is typically longer when all three types of UFL are treated simultaneously but this has not yet been fully demonstrated in clinical studies
Is there any evidence that higher doses can improve efficacy and duration of response?	Trial data show that higher doses (above those currently licensed) can further improve GL treatment outcomes without affecting safety [16, 30, 37] Additional studies are needed to assess the effectiveness and safety of higher doses in the treatment of LCL and FHL
Is there a ceiling dose?	Some data suggest a ceiling dose; however, this is not yet completely clear and may differ by indication, patient sex, age, and muscle mass [16, 30, 37]
Does increasing the dose of onabotulinumtoxinA affect safety outcomes?	There is no evidence of a link between onabotulinumtoxinA dose and the incidence or severity of any category of AEs [16, 28, 29, 30, 37] Underdosing does not appear to reduce the risk of AEs but could limit effectiveness—so it may be advisable to avoid going below the licensed dose unless there is a clear clinical rationale

Abbreviations: AE, adverse event; BoNT, botulinum neurotoxin; FHL, forehead lines; GL, glabellar lines; LCL, lateral canthal lines; SV2, synaptic vesicle glycoprotein 2; UFL, upper facial lines.

Comparison of clinical duration analyses between studies is complicated by the lack of a single comprehensive definition. However, trial data with the standard onabotulinumtoxinA dose of 20 U for GL suggest a median duration of effect among responders of ~4 months (based on response maintenance at maximum contraction) [34].

Dose‐ranging studies of onabotulinumtoxinA have important implications for clinical practice. They show that treating UFL with onabotulinumtoxinA at below the approved dose is associated with inferior outcomes—including reduced response rates, shorter duration of effect, and lower patient satisfaction [28, 31, 32, 33]. Furthermore, there is no evidence that the risk of AEs is decreased by underdosing onabotulinumtoxinA in these indications.

The doses assessed in robust registrational studies have proven safety and efficacy. Practitioners should adopt EBM as their starting point for decision making (considering both trial data and real‐world evidence), and only use lower doses when they judge there to be a clear clinical rationale.

By contrast, both early and recent evidence suggest that it may be possible to further improve GL treatment outcomes, without affecting the safety profile, using doses above those currently licensed [16, 30, 37]. However, clinical judgment should always be applied when determining the optimal dose for individual patients. Additional trials are needed to assess whether similar results can be achieved with higher doses in the treatment of LCL and FHL. Further studies are also required to examine potential ceiling doses and to better understand the dose–response relationship in specific patient subgroups.

These gaps in current knowledge represent an important constraint that should be addressed in the coming years. We also acknowledge that the current paper is a narrative rather than a systematic review, which some readers may consider to be a limitation—although the author group is highly experienced and indeed several were investigators in many of the key studies cited.

To conclude, from a practical perspective, appropriate dosing of onabotulinumtoxinA is essential to maximizing effectiveness and duration in aesthetic indications—and hence for enhancing patient satisfaction with results.

## Author Contributions

All authors were involved in the interpretation of data for the work, drafting of the manuscript, and revising the manuscript critically for important intellectual content; gave final approval of the version to be published; and agreed to be accountable for all aspects of the work.

## Ethics Statement

The authors have nothing to report.

## Conflicts of Interest

Wolfgang Philipp‐Dormston is a consultant, speaker, and clinical trial investigator, and has received scientific grants and honoraria, from AbbVie, Evolus, Galderma, and Merz. John Joseph is a speaker and/or consultant for AbbVie, Evolus, Galderma, Merz, and Revance; has received research funding from AbbVie, Croma, Galderma, Merz, Teoxane, and Evolus; and owns stocks in Alpheon, Evolus, and Revance. Jean Carruthers is a consultant and investigator for Acorn, Alastin, Appiell, AbbVie, Avari, Bonti, Del Nova, Evolus, Fount Bio, In Mode, Inverse Genomic, Jeune Aesthetics, Merz, Object Pharma, Revance, and Sofwave. John Fezza is a consultant, trainer and speaker for AbbVie, a consultant and trainer for Revance, an advisor for Evolus, and a speaker for RVL. Mansi Mukherjee is a consultant and speaker for AbbVie. Ahmed Yasin and Maria Musumeci are employees of AbbVie Inc. and may own stock/stock options in the company.

## Data Availability

Data sharing is not applicable to this article as no datasets were generated or analyzed. AbbVie is committed to responsible data sharing regarding the clinical trials we sponsor. This includes access to anonymized, individual, and trial‐level data (analysis data sets), as well as other information (e.g., protocols, clinical study reports, or analysis plans), as long as the trials are not part of an ongoing or planned regulatory submission. This includes requests for clinical trial data for unlicensed products and indications. These clinical trial data can be requested by any qualified researchers who engage in rigorous, independent, scientific research, and will be provided following review and approval of a research proposal, Statistical Analysis Plan (SAP), and execution of a Data Sharing Agreement (DSA). Data requests can be submitted at any time after approval in the US and Europe and after acceptance of this manuscript for publication. The data will be accessible for 12 months, with possible extensions considered. For more information on the process or to submit a request, visit the following link: https://vivli.org/ourmember/abbvie/ then select “Home.”
